# Reactivity Trends of Lewis Acidic Sites in Methylaluminoxane and Some of Its Modifications

**DOI:** 10.1021/acs.inorgchem.0c00533

**Published:** 2020-04-09

**Authors:** Francesco Zaccaria, Peter H. M. Budzelaar, Roberta Cipullo, Cristiano Zuccaccia, Alceo Macchioni, Vincenzo Busico, Christian Ehm

**Affiliations:** † Dipartimento di Scienze Chimiche, Università di Napoli Federico II, Via Cintia, 80126 Napoli, Italy; ‡ Dipartimento di Chimica, Biologia e Biotecnologie and CIRCC, Università di Perugia, Via Elce di Sotto 8, 06123 Perugia, Italy

## Abstract

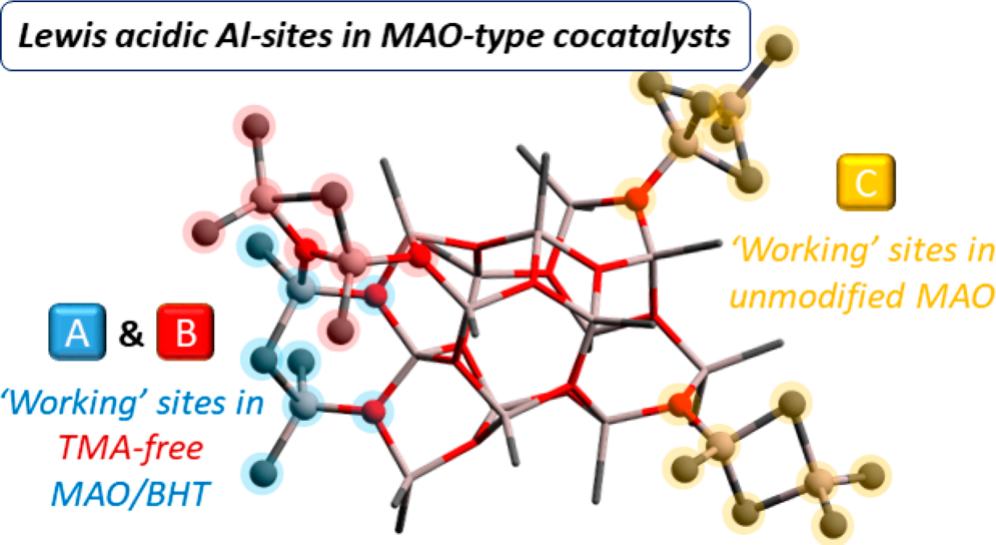

The established model cluster (AlOMe)_16_(AlMe_3_)_6_ for methylaluminoxane (MAO) cocatalyst has been studied by density functional theory, aiming to rationalize the different behaviors of unmodified MAO and TMA-depleted MAO/BHT (TMA = trimethylaluminum; BHT = 2,6-di-*tert*-butyl-4-methylphenol), highlighted in previous experimental studies. The tendency of the three model Lewis acidic sites **A**–**C** to release neutral Al fragments (i.e., AlMe_2_R; R = Me or bht) or transient aluminum cations (i.e., [AlMeR]^+^) has been investigated both in the absence and in the presence of neutral N-donors. Sites **C** are most likely responsible for the activation capabilities of TMA-rich MAO, but TMA depletion destabilizes them, possibly inducing structural rearrangements. The remaining sites **A** and **B**, albeit of lower Lewis acidity, should be still able to release cationic Al fragments when TMA-depleted modified MAOs are treated with N-donors (e.g. [AlMe(bht)]^+^ from MAO/BHT). These findings provide tentative interpretations for earlier observations of donor-dependent ionization tendencies of MAO and MAO/BHT and how TMA depleted MAOs can still be potent activators.

## Abstract 2

A model cluster for methylaluminoxane (MAO) was used to study the tendency of model Lewis acidic sites to release neutral Al fragments or transient aluminum cations both for unmodified MAO and TMA-depleted MAO/BHT (TMA = trimethylaluminum; BHT = 2,6-di-*tert*-butyl-4-methylphenol). Albeit only sites of lower Lewis acidity are found in TMA depleted modified MAOs, they can release cationic Al fragments when treated with certain N-donors. This shows how TMA depleted MAOs can still be potent activators.

## Introduction

Methylaluminoxane (MAO)^1,2^ is typically used as a cocatalyst in molecular olefin polymerization catalysis for the industrial production of performance polymers,^3−5^ due to its remarkable combination of good impurity scavenging properties and excellent alkylating and abstracting capability.^1,2,6,7^ Moreover, MAO can be used to heterogenize molecular catalysts on supports like silica or alumina.^2,8,9^

Since its serendipitous discovery in the 1980s,^2^ MAO is typically produced via controlled hydrolysis of trimethylaluminum (TMA), leading to a rather complex mixture of species.^1,2,6,7^ Great efforts have been devoted to the structural elucidation of this aluminoxane, but the task remains—despite some considerable advancements—largely unaccomplished. The crystal structure of its higher homologue *tert*-butylaluminoxane (TBAO), obtained by controlled hydrolysis of tri-*tert*-butylaluminum, has been determined, featuring well-defined (AlO*t*Bu)_*n*_ cages with four-coordinate Al and three-coordinate O centers at the edges of four- or six-membered rings.^10^ Experimental and computational studies have shown that similar cages of pure (AlOMe)_*n*_ are not stable enough. In the absence of the bulky *t*Bu groups, rearrangements maximize the number of the less strained six-membered faces.^11−25^

Besides, some residual TMA from the synthesis (typically accounting for up to 1/3 of the total Al content)^22,26^ likely serves a structural, stabilizing role in MAO clusters. It is generally accepted that hydrolysis of TMA yields a complex and dynamic distribution of (AlOMe)_*n*_(AlMe_3_)_*m*_ cages.^1,11−25^ The formula accounts indiscriminately for formal AlMe_3_ that is actually incorporated into the cage backbone and for proper AlMe_3_ molecules that are reversibly associated with the Al-clusters. The latter serve to stabilize residual unsaturated Al and O atoms (“structural” AlMe_3_) and are known to undergo exchange equilibria with residual “free” TMA molecules.^11,14,24,26−28^

These dynamic equilibria are particularly relevant in MAO modifications involving TMA depletion. This can be achieved, for instance, by vacuum drying (bp of TMA = 125 °C)^29−31^ or by addition of suitable scavengers like 2,6-di-*tert*-butyl-4-methylphenol (BHT, vide infra).^32−34^ In all cases, “free” TMA is likely removed/scavenged first, consequently inducing a partial release of “structural” AlMe_3_ molecules. This dissociation exposes unsaturated Al and/or O atoms on cage edges, destabilizing the cluster and inducing structural rearrangements. Formation of larger Al-cages, upon TMA depletion, has been observed experimentally by diffusion NMR spectroscopy^29,33^ and cryoscopy.^27^

**Scheme 1:**
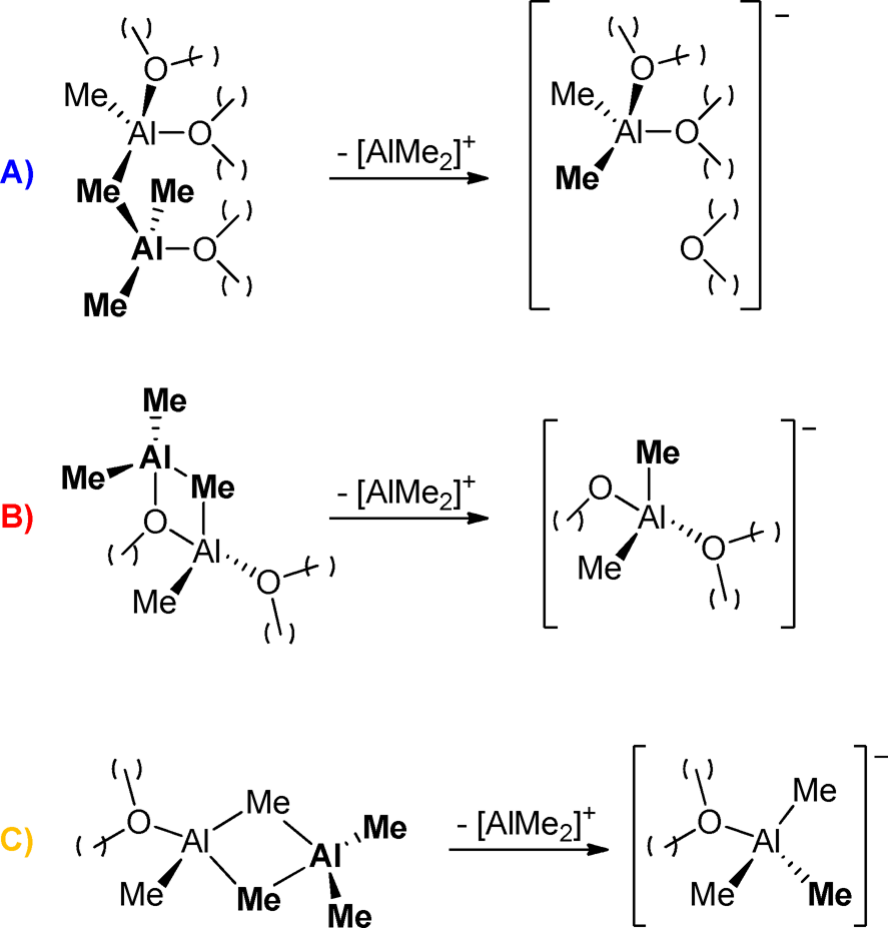
Generation of AlMe_2_^+^ species from Type **A**–**C** Sites in MAO See also Figure 1.

Importantly, in addition to their stabilizing role, “structural” AlMe_3_ molecules are proposed to serve functional roles. In particular, they are thought to be responsible for the Lewis acidity of MAO via generation of transient [AlMe_2_]^+^ species relevant for precatalyst activation (Scheme 1).^18,26,35−38^ Experimentally, this type of reactivity has been explored via the interaction of MAO with small amounts of neutral O- or N-donors (4–10 mol %).^26,39−43^ Bipyridine (**bipy**), for instance, extracts [AlMe_2_]^+^ from MAO with high chemoselectivity in the form of the donor stabilized adduct [AlMe_2_(bipy)]^+^.^41^

Similarly, pyridine (**py**), can generate the corresponding [AlMe_2_(py)_2_]^+^ adduct, but concomitant formation of neutral AlMe_3_(py) is also observed.^26,28,41^

Recently, analogous reactions have been reported for the phenol-modified MAO/BHT.^44^ It has been proposed that addition of the Brønsted acidic BHT to MAO leads to (a) conversion of “free” TMA and a fraction of “structural” AlMe_3_ into the relatively inert AlMe(bht)_2_ complex via methane generation^45,46^ and (b) conversion of the remaining “structural” AlMe_3_ in analogous “structural” AlMe_2_(bht) molecules, resulting in [(AlOMe)_0.87_(AlMe_2_ bht)_0.13_]_*n*_ cages (bht = BHT phenolate).^29,33,44^ Addition of **bipy** to MAO/BHT leads to the formation of [AlMe(bht)(bipy)]^+^, while **py** selectively generates neutral AlMe_2_(bht)(py).^44^

The interaction with **py** has been studied also for MAO supported on silica;^36,47^ Brønsted acidic Si–OH groups of silica are known to scavenge TMA similarly to BHT.^2,8,36,47−49^ However, the complexity of these heterogeneous systems hampers straightforward connections with the structure and reactivity of unmodified MAO in solution.^50^

**Figure 1:**
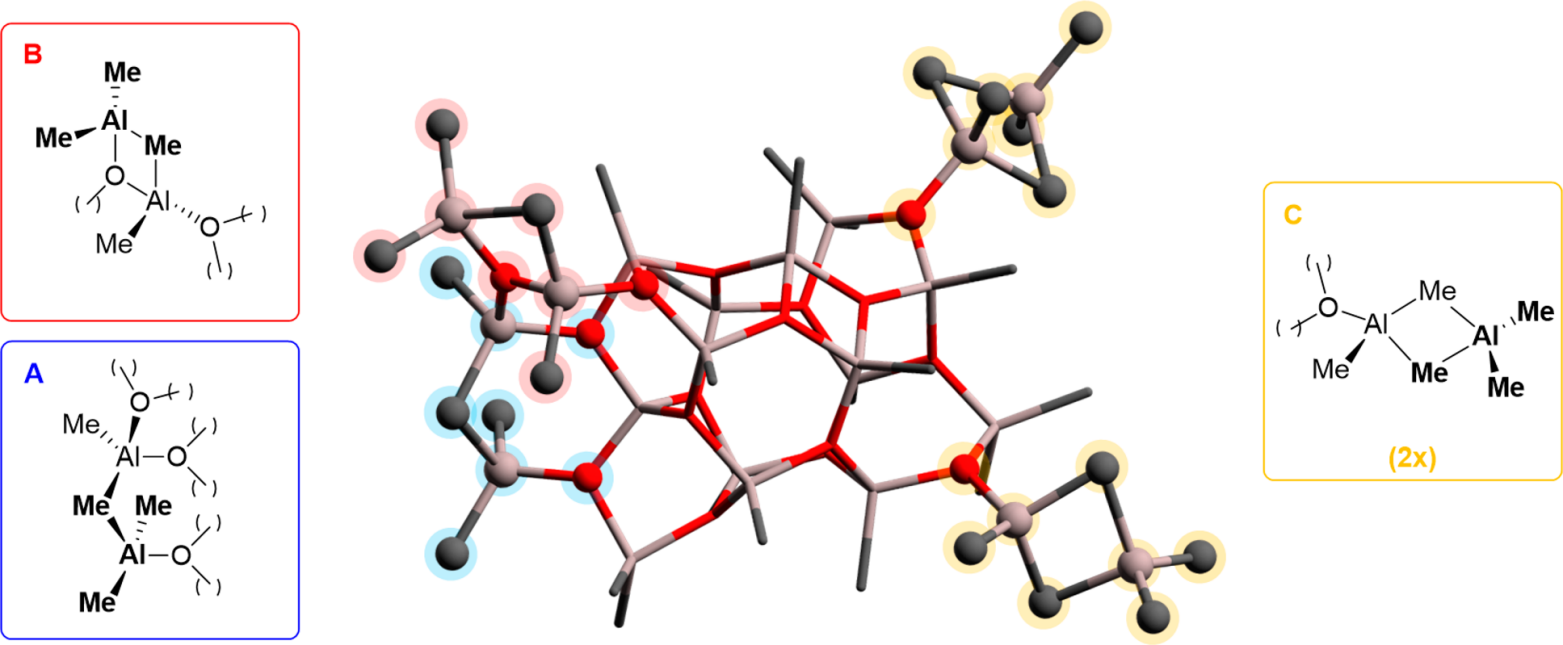
DFT optimized structure of **16,6**, proposed by Linnolahti and co-workers as the most stable cage for MAO (O atoms in red, Al atoms in gray and C atoms in black, H atoms omitted for clarity).^16,19^ “Structural” TMA molecules, binding different Al-sites (one type **A** and **B**, and two type **C**) of the cage backbone, are highlighted.

In extensive computational studies, Linnolahti and co-workers^16,19^ have proposed that the cage **16,6** (i.e., having *n* = 16 and *m* = 6; Figure 1a)^19,51^ represents the most stable model MAO cluster, in line with several other theoretical^13,52,53^ and spectroscopic findings.^26,33,40,42,43,54,55^ This **16,6** cage features four “structural” AlMe_3_ molecules in three different cluster environments, **A**, **B**, and **C** (emphasized by different colors in Figure 1).^37^ Assuming cage **16,6** as a realistic model for unmodified MAO, the reactivity of the proposed Lewis acidic sites **A**–**C** has been explored in some density functional theory (DFT) studies, for example, identifying **C** type sites as those being more prone to release [AlMe_2_]^+^ and AlMe_3_.^37,55^

In the present paper, a systematic computational study is reported, aiming to provide further insights in the functional roles carried out by the Lewis acidic Al-sites. The representative model cluster **16,6** was considered for MAO, along with its bht-decorated analogues representing MAO/BHT. The thermodynamics of [AlMeR]^+^ and/or AlMe_2_R dissociation (R = Me or bht) was investigated both in the absence and in the presence of neutral N-donors like **py** and **bipy**, providing a close comparison with previously reported experimental studies.^26,28,41,44^

## Results

The computational protocol M06-2X/TZ(PCM)//TPSSTPSS/DZ has been previously benchmarked^56,57^ and widely used for modeling of Al-species relevant to olefin polymerization^33,44,58,59^ and other research fields,^60^ and it has been shown to be suitable for studies related to MAO activators.^19,33,35,41,51,61^ Additional inclusion of Grimme-type long-range dispersion corrections^62,63^ does not affect computational results significantly (see Supporting Information).

**Scheme 2:**
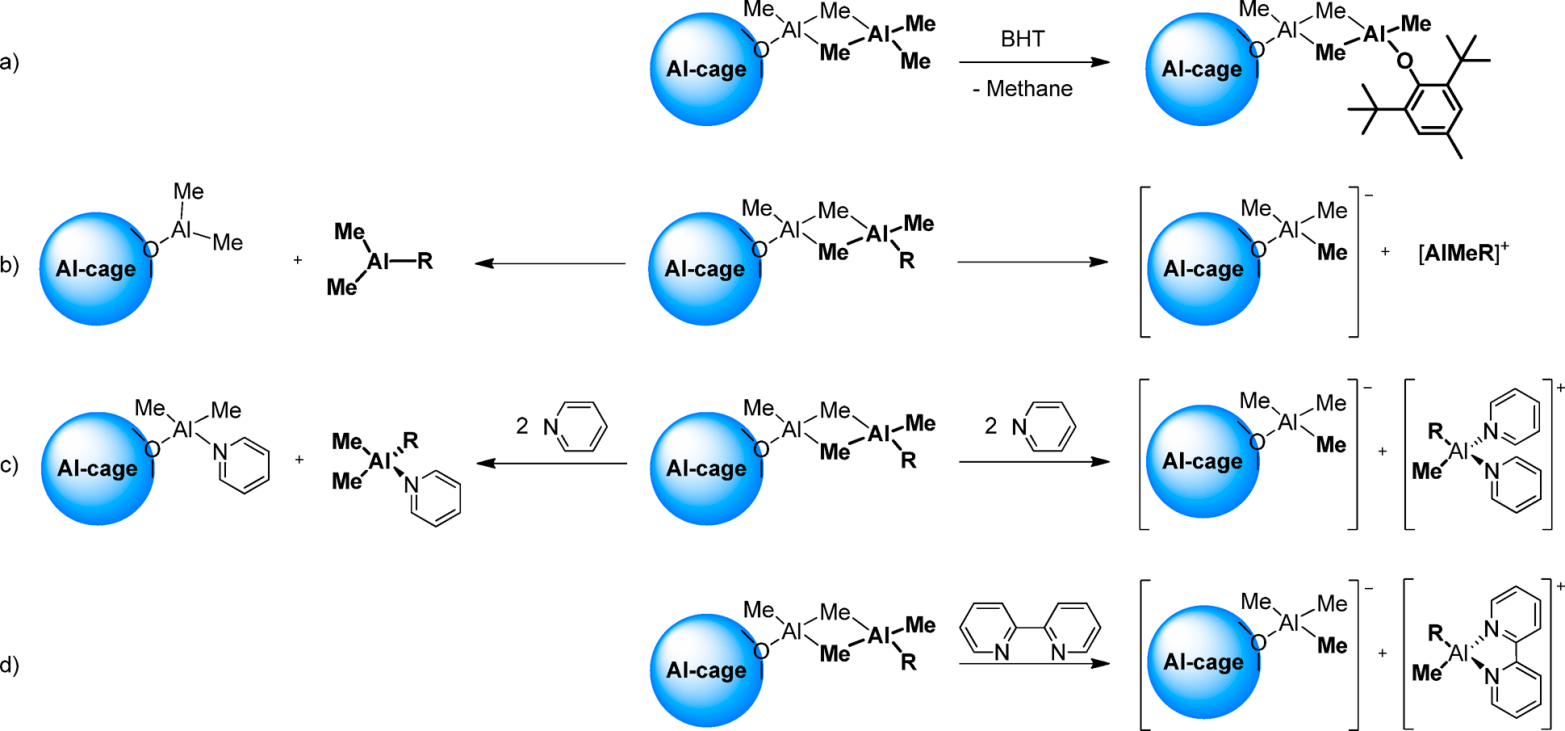
Reactions Studied in This Work (a) Replacement of “structural” AlMe_3_ with “structural” AlMe_2_(bht); (b) release of [AlMeR]^+^ or AlMe_2_R in the absence of neutral donors; (c) release of [AlMeR]^+^ or AlMe_2_R in the presence of **py**; (d) release of [AlMeR]^+^ in the presence of **bipy**. Type C Lewis acidic sites used as exemplifying case for the three sites shown in Figure 1. See analogous Schemes S1 and S2 in the Supporting Information for **A** and **B** type sites. “Structural” AlMe_2_R molecules highlighted in bold (R = Me or bht).

Scheme 2 summarizes the reactions studied in this work, using type **C** site as representative example (see Supporting Information for the corresponding schemes for **A** and **B**):

- Replacement of “structural” AlMe_3_ with the analogous AlMe_2_(bht) (Scheme 2a)
- Formation of [AlMeR]^+^ or AlMe_2_R in the absence of donors (Scheme 2b)
- Formation of [AlMeR]^+^ or AlMe_2_R in the presence of neutral donors like **py** (Scheme 2c) or **bipy** (Scheme 2d). In the latter case, only ionization is modeled. Extraction of neutral fragments would lead to the unfavorable formation of five-coordinate AlMe_2_R(bipy) species^41,44^

It should be noted here, that the experimentally found MAO anion with the highest detected abundance is also a **16,6** cluster,^42^ which among other potential routes can be formally derived from a **16,7** neutral cluster via [AlMe_2_]^+^ release, a **16,6** cluster via methide abstraction, or a **16,6** cluster via [AlMe_2_]^+^ abstraction to form **16,5** followed by TMA coordination to reform **16,6**. The structure of the anionic **16,6** cluster is not identical with a neutral **16,6** cluster nor is the neutral **16,6** cluster considered here able to accept a methide without rearrangements. Due to the complex equilibria between different MAO clusters, we therefore decided to focus on the *most abundant neutral species* identified by Linnolahti.^16,19^ Reactions of interest are modeled neglecting any structural rearrangements that might consequently occur. The behavior of each Al-site type should be rather independent from the cage size; previous extensive DFT studies^19,37^ highlighted that *structural characteristics of the edge sites are independent of the sizes, forms, and shapes of the MAOs*.^19^ Corroborating this, nearly identical behaviors (within 1.5 kcal/mol) are predicted for the two inequivalent **C**-type sites of **16,6** studied here.

DFT estimated reaction Gibbs free energies (Δ*G*_R_) are reported in Tables 1 and 2. These values are only intended for semiquantitative comparison among the different acidic sites, since the complexity of the systems likely hampers the accurate identification of true global minima.^64^ Furthermore, reactions are modeled assuming naked ions in a solvent continuum, rather than the ion pairs typically formed in the low polarity solvents used (see below for further discussion on this point).^7,65,66^

**Table 1:** Calculated Δ*G*_R_ (at 298 K, in kcal/mol) for the Reaction of “Structural” AlMe_3_ with BHT Leading to “Structural” AlMe_2_(bht) Incorporation (Scheme 2a)

entry	Al site	Δ*G*_R_
1	**A**	–23.8
2	**B**	–30.1
3	**C**	–27.8

**Table 2:** Calculated Δ*G*_R_ (at 298 K, in kcal/mol) for the Release of [AlMeR]^+^ or AlMe_2_R from MAO or MAO/BHT (R = Me or bht; see Scheme 2b–d)

entry	Al site	AlMe_3_ loss	AlMe_2_(bht) loss	[AlMe_2_]^+^ loss	[AlMe(bht)]^+^ loss
without donors (Scheme 2b)					
1	**A**	24.6	14.7	104.4	91.6
2	**B**	35.9	32.3	96.8	90.2
3	**C**	7.9	2.3	75.6	67.8
with **py** (Scheme 2c)					
4	**A**	0.8	–8.0	23.5	18.0
5	**B**	–8.1	–10.7	15.9	16.7
6	**C**	–34.1	–38.6	–5.3	–6.8
with **bipy** (Scheme 2d)					
7	**A**	–	–	21.1	16.3
8	**B**	–	–	13.5	15.0
9	**C**	–	–	–7.7	–8.5

The Δ*G*_R_ values reported in Table 1 for conversion of “structural” AlMe_3_ into “structural” AlMe_2_(bht), upon addition of BHT to MAO, clearly indicate a highly exergonic process for all the various types of acidic sites (Scheme 2a). These data support the hypothesis that no residual “structural” AlMe_3_ should be present on MAO/BHT-type cages, provided that enough BHT is added.^33^

The results concerning the generation of neutral or ionic fragments are reported in Table 2 (see also Scheme 2b–d). The reactions in the absence of stabilizing donors have been previously explored by DFT only for unmodified MAO (vide infra).^37,55^ Here, the release of “structural” AlMe_3_ is calculated to be an endergonic process for all sites with decoordination energies rising in the order **C** ≪ **A** < **B** (Table 2, entries 1–3). The decoordination energy of “structural” AlMe_3_ from **16,6** via site **C** (≈8 kcal/mol) is quite small and comparable to the dimerization energy of TMA (6–7 kcal/mol);^56,67^ appreciably higher Δ*G*_R_ are estimated for sites **A** and **B** (25 and 36 kcal/mol, respectively), instead. The peculiarity of sites **C** lies in the “structural” AlMe_3_ molecules being bound to the Al site of the cage via two bridging methyl groups (similar to the TMA dimer), leading to a more labile interaction compared to sites **A** and **B** with one bridging methyl and a proximal two-coordinate O atom on the cage edge (Figure 1; compare also Scheme 1b with Schemes S1b and S2b).^37^

**Scheme 3:**
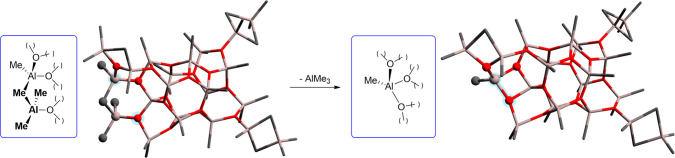
Formation of a New Al–O Bond between the Unsaturated Al and O Atoms (highlighted in blue) Generated upon AlMe_3_ Dissociation from Site **A** O atoms in red, Al atoms in grey and C atoms in black, H atoms omitted for clarity.

The specific structure of site **A**, in which the Al and O atoms involved in the interaction with AlMe_3_ are adjacent but not bound to each other (Figure 1), leads, upon AlMe_2_R detachment, to unsaturated Al and O atoms that are spatially close enough to form a new Al–O bond (Scheme 3). This is associated with small local rearrangements of the cage structure, and is not restricted to **16,6** but could also occur in other MAO clusters. The formation of this bond exemplifies the complexity of the dynamic behavior of MAO^1^ and partially compensates the energy loss due to TMA detachment, explaining the lower Δ*G*_R_ of about 11 kcal/mol estimated for **A** with respect to **B** (Table 2).

AlMe_2_(bht) is bound slightly weaker than AlMe_3_ to **B** and **C** (ΔΔ*G*_R_ ≈ 4–6 kcal/mol; Table 2, entries 1–3) and especially to **A** (ΔΔ*G*_R_ ≈ 10 kcal/mol). As shown in Table S3 and Figure S1, the bht fragments of “structural” AlMe_2_(bht) are rotated out of the optimal Al–O–C_bht_ binding geometry, likely due to steric congestion. This distortion is most prominent for site **A** and might be (co)responsible for the particularly weaker binding of the bulky Al-phenolate fragment to this site.

Similar trends to those highlighted for AlMe_2_R are calculated for formation of cationic [AlMeR]^+^, that is, ionization should occur more easily for **C** than for **A** and **B**. [AlMe(bht)]^+^ should be released somewhat more easily than [AlMe_2_]^+^ from each site (Table 2, entries 1–3). The higher propensity of sites **C** to release AlMe_3_ and [AlMe_2_]^+^ is in line with previous reports by Linnolahti and co-workers on unmodified MAO.^37,55^ The presence of Al-sites having different Lewis acidity has been highlighted also by some experimental studies.^54,68,69^

**Figure 2:**
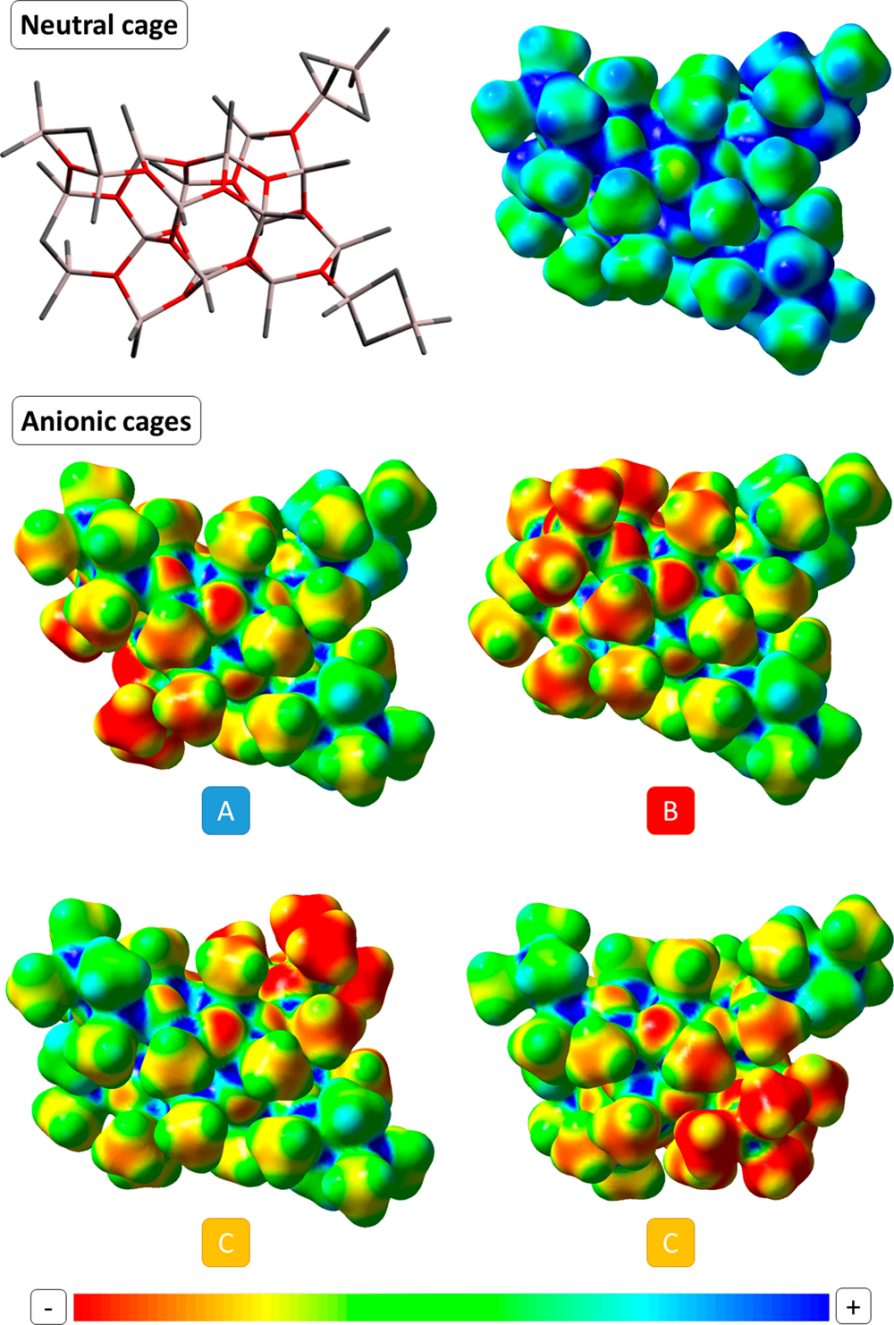
Mapped electrostatic potential surface from total electron density for the neutral **16,6** cage (top) and the anionic cages generated therefrom upon [AlMe_2_]^+^ dissociation from sites **A**–**C** (bottom). Structure of **16,6** provided for comparison (H omitted for clarity; see also Figure 1 and Experimental Section).

Upon ionization, the negative charge appears to be delocalized, albeit not over the whole cage, especially for sites **A** and **B** (Figure 2 and Table S4). Charge dissipation is known to be crucial for generating weakly coordinating anions and, consequently, highly active catalysts.^6,7^ The ability of MAO to generate large anions with a delocalized negative charge has been proposed as one of the origins of the excellent cocatalytic properties of this aluminoxane.^6,7,70,71^ In the presence of a neutral monodentate donor like **py**, all reactions are predicted to be significantly easier (Table 2, entries 4–6). The dissociation of AlMe_3_ becomes exergonic for **B** (−8 kcal/mol) and particularly for **C** (−34 kcal/mol), and nearly energetically neutral for **A**. Release of AlMe_2_(bht) is again favored with respect to that of AlMe_3_ for each site (ΔΔ*G*_R_ ≈ 5–7 kcal/mol). The exergonicity is enforced by two main contributions. First, **py** stabilizes the leaving molecular species AlMe_2_R by forming coordinately saturated AlMe_2_R(py) complexes. Second, **py** can bind also to the three-coordinate Al atoms on the cage edge generated by the dissociation, therefore preventing cage destabilization (compare reactions in Scheme 2b,c, left paths).^72^ The former contribution is cage independent, while the latter varies depending on the Lewis acidic site involved. Indeed, trends in relative Δ*G*_R_ estimated for donor coordination reflect those in relative ΔΔ*G*_R_ for AlMe_2_R detachment in the absence and presence of **py**, as reported in Table S5. The cage stabilization due to **py** coordination is only in the order of ∼6 kcal/mol for **A**, while it is much higher for **B** and **C** (about 26 and 24 kcal/mol, respectively). The appreciably lower Δ*G*_R_ in the former case is due to the formation of the aforementioned Al–O bond (Scheme 3), which represents an alternative albeit milder stabilization route for the unsaturated atoms generated upon AlMe_2_R dissociation from **A**.

Also ionization is facilitated by the presence of **py** (Table 2, entries 4–6), up to the point that it is calculated to be exergonic for sites **C** (−5 kcal/mol for [AlMe_2_]^+^ and −7 kcal/mol for [AlMe(bht)]^+^) but not for sites **A** (>16 kcal/mol) and **B** (>18 kcal/mol). Here, four-coordinate Al atoms are generated on the cage, and only the stabilization of the [AlMeR]^+^ fragments by two **py** molecules provides an increased driving force (Scheme 2c, right path). Different from **B**, sites **A** and **C** generate [AlMe(bht)]^+^ more easily than [AlMe_2_]^+^.

The same trends are predicted for ionization in the presence of the bidentate **bipy** (Table 2, entries 7–9). The stronger binding of this bidentate donor to cationic aluminum is responsible for a further drop of predicted Δ*G*_R_ by a few kcal/mol compared to **py**.

## Discussion

**Figure 3:**
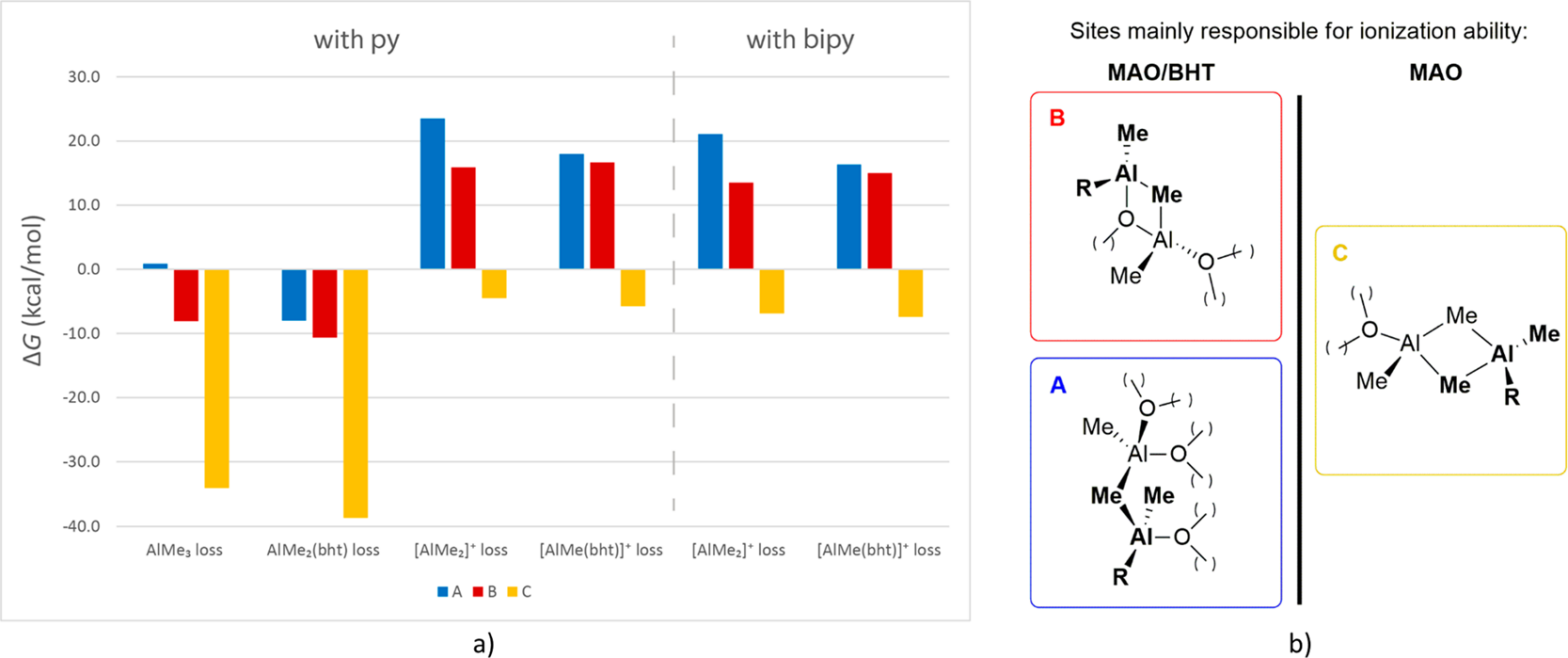
(a) Graphical comparison of calculated Δ*G*_R_ at 298 K (in kcal/mol) for the release of [AlMeR]^+^ or AlMe_2_R from MAO or MAO/BHT (R = Me or bht; see also Table 2). (b) Structures of sites **A**–**C** relevant for MAO and MAO/BHT ionization properties.

These results clearly indicate that sites **A**–**C** are expected to behave quite differently. Thus, considering these three as realistic models for Lewis acidic sites, the data presented here allow for some interpretation of the issues related to the properties of MAO and its modifications outlined in the Introduction. The following discussion will mainly focus on Δ*G*_R_ calculated for the reactions in the presence of donors, graphically summarized in Figure 3a, since they provide a more straightforward comparison with experimental results. The features of MAO are discussed first, followed by those of MAO/BHT.

### Sites Responsible for MAO Activation Properties

It is generally accepted that only one or slightly more than one acidic site per cage should be working, on average.^12,33,54,73^ Type **C** sites are those releasing [AlMe_2_]^+^ fragments most easily and therefore likely responsible for the abstracting capability of MAO (Figure 3). This is in line with previous reports that considered ionization only in the absence of donors.^37^

### Sites Inducing Structural Rearrangements of MAO Cages upon TMA Depletion

Type **C** sites also tend to release “structural” AlMe_3_ most easily (Figure 3). Thus, TMA depletion by either vacuum drying or addition of BHT should expose and, consequently, destabilize these sites, inducing Al-cage rearrangements. Modified TMA-free MAOs, like MAO/BHT, should therefore contain mostly acidic sites of type **A** and **B**.

### Sites Binding “Structural” AlMe_2_(bht) in MAO/BHT

Sites **A** and **B** are predicted to bind “structural” AlMe_2_R rather strongly (Figure 3). Therefore, upon addition of BHT, “structural” AlMe_3_ molecules bound to these sites in MAO should be those transformed into “structural” AlMe_2_(bht) without being released by the Al cages.

### Sites responsible for MAO/BHT activation properties

**Scheme 4:**
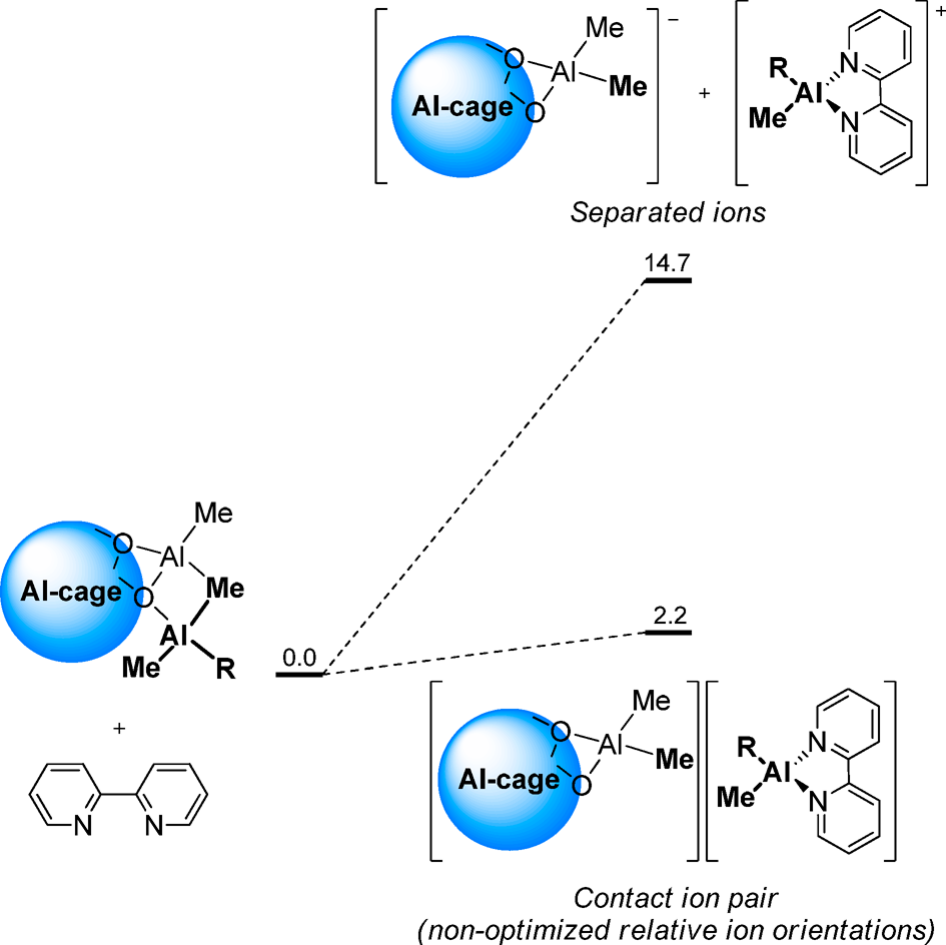
Comparison of Calculated Δ*G*_R_ at 298 K (in kcal/mol) for the Release of [AlMe(bht)(bipy)]^+^ from Site **B** of MAO/BHT within the Naked Ion Approximation or Considering a Contact Ion Pair (R = Me or bht)

Based on the above considerations, DFT results suggest that MAO/BHT contains mostly sites **A** and **B**, and ionization is predicted to be similarly thermodynamically unfavorable in both cases (Figure 3). However, it is worth noting that the calculated absolute Δ*G*_R_ for ionization is likely overestimated. Ion pair formation occurs in the low polarity solvents used experimentally,^7,65^ which, as previously demonstrated, can account for tens of kcal/mol,^74^ but it was not modeled here (vide supra). To prove this concept, the representative ion pair [AlMe(bht)(bipy)]^+^[**16,5**]^−^ has been modeled (fully relaxed) for **B**, assuming that the cation remains in the proximity of the Al site it was generated from. Calculated Δ*G*_R_ drops from 15.0 kcal/mol for the isolated ions to only 2.2 kcal/mol for the contact ion pair (Scheme 4). A Δ*G*_R_ close to zero for this reaction is consistent with the experimental observation that ionization occurs with **bipy** but not **py**.^44^

### Why does **py** abstract [AlMe_2_]^*+*^ form MAO but not [AlMe(bht)]^*+*^ from MAO/BHT?

Computational results indicate that, for a given site, generation of [AlMe_2_]^+^ or [AlMe(bht)]^+^ should be similarly easy. However, as discussed in the previous sections, sites that release [AlMe_2_]^+^ in MAO (**C**) should not be of the same type as those releasing [AlMe(bht)]^+^ in MAO/BHT (**A/B**). The latter type is appreciably less prone to undergo ionization (Figure 3), which can explain why addition of an N-donor like **py** leads to (partial) ionization of MAO but not MAO/BHT.^44^ The difference between the two cocatalysts and the different Lewis acidic sites becomes experimentally less evident with the bidentate donor **bipy**, which is a more effective ionizing agent.

## Conclusions

The structure and reactivity of MAO activators are known to be critically dependent on their Lewis acidic sites. In this work, the thermodynamics of [AlMeR]^+^ or AlMe_2_R dissociation from some established model MAO cages was systematically investigated by DFT. Different from previous reports by others,^37,55^ not only the reactivity of unmodified MAO but also that of TMA-free MAO/BHT was considered. Furthermore, the reactions were modeled both in the absence and in the presence of neutral N-donors, in the latter case providing a direct connection with known experimental observations.^26,39−43^ An interpretation of how modifications of MAO can affect acidic sites composition and, consequently, cocatalytic properties is proposed.

In unmodified MAO, type **C** sites should be those releasing [AlMe_2_]^+^ and AlMe_3_ most easily. This implies that (a) they are likely responsible for the activating capability of MAO and (b) the “structural” AlMe_3_ molecules bound to these sites are largely released upon TMA depletion (e.g., by vacuum drying or reaction with Brønsted acids like BHT). The consequent liberation of unsaturated three-coordinate Al-atoms on the cage can lead to structural rearrangements that “annihilate” these sites.

In TMA-depleted MAOs, “structural” AlMe_2_R molecules should therefore be bound primarily to the remaining sites **A** and **B**. In the case of MAO/BHT, calculations indicate that the two sites have similar tendencies to release [AlMe(bht)]^+^ and AlMe_2_(bht). Sites **A** and **B** should therefore be responsible for the properties of MAO/BHT, even though their reactivity is appreciably lower than that of sites **C** in unmodified MAO. These computational results explain the lower propensity of MAO/BHT with respect to MAO to undergo ionization upon interaction with **py**:^44^ this depends not only on the different stability/electrophilicity of [AlMe(bht)]^+^ vs [AlMe_2_]^+^ cations but also on the reactivity of **B** vs **C**-type sites they originate from.

The reactivity trends of type **A**–**C** sites should be rather independent of the size of cage they belong to, since their properties are mainly determined by their local structures. Importantly, the apparent diversity of Lewis acidic sites in MAO and its TMA depleted counterparts indicates that strongly Lewis acidic Al-sites like **C** are not necessarily a prerequisite for an effective abstractor.

## Experimental Section

Following the protocol described in ref (57), all geometries were fully optimized using the Gaussian 09 software package^75^ in combination with the OPTIMIZE routine of Baker^76,77^ and the BOpt software package.^78^ All relevant structures were fully optimized at the TPSSTPSS level^79^ of theory employing correlation-consistent polarized valence double-ζ Dunning(DZ) basis sets (cc-pVDZ quality)^80,81^ from the EMSL basis set exchange library.^82^ All calculations were performed at the standard Gaussian 09 quality setting [Scf = Tight and Int(Grid = fine)]. Final single-point energies and natural bond orbital^83^ analyses were calculated at the M06-2X level of theory^84^ employing triple-ζ Dunning (TZ) basis sets (cc-pVTZ quality).^80^ Solvent corrections where included at this stage by the polarized continuum model (PCM);^85^ a typical solvent used in olefin polymerization, namely toluene, was considered. The density fitting approximation (resolution of identity) was used at the optimization stage as well as for final energy calculations.^86−89^ Enthalpies and Gibbs free energies were then obtained from TZ single-point energies and thermal corrections from the TPSSTPSS/cc-pVDZ-(PP) vibrational analyses; entropy corrections were scaled by a factor of 0.67 to account for decreased entropy in the condensed phase.^90−92^ The structure of **16,6**, optimized by Linnolahti and co-workers, was used as starting the geometry.^16,19^ Electrostatic potential maps of Figure 2 were generated from cube files for electrostatic potential and electron density using the GaussView software (isosurface value = 0.01). The cubes were obtained by means of the *cubegen* routine of Gaussian using the formatted checkpoint files of M06-2X single point energy calculations.

## Supporting Information Available

Coordinates for optimized geometries (XYZ)
Additional DFT results (DFT results including long-range Grimme dispersion corrections, details for steric congestion at Al-bht sites, reaction schemes for sites **A** and **B**, ranges of NPA charges, Gibbs free energies for py coordination after AlMe_2_R dissociation, final energies, entropy and enthalpy corrections, full Gaussian citation (PDF)

## Supplementary Material

ic0c00533_si_001.xyz
