# Supplementary material for: Reactivity Trends of Lewis Acidic Sites in Methylaluminoxane and Some of Its Modifications
Source: Inorg Chem. 2020 Apr 9;59(8):5751–9. doi: 10.1021/acs.inorgchem.0c00533 (PMC7997381; doi:10.1021/acs.inorgchem.0c00533)
Supplement: Supplementary file 2 — ic0c00533_si_002.pdf [file ic0c00533_si_002.pdf]

Supporting Information

Reactivity Trends of Lewis Acidic Sites in Methylaluminoxane and Some of Its Modifications

Francesco Zaccaria,[a,b] Peter H. M. Budzelaar,[a] Roberta Cipullo,[a] Cristiano Zuccaccia,[b] Alceo Macchioni,[b] Vincenzo Busico[a] and Christian Ehm[a,]*

[a] Dipartimento di Scienze Chimiche, Università di Napoli Federico II, Via Cintia, 80126 Napoli, Italy; [b] Dipartimento di Chimica, Biologia e Biotecnologie and CIRCC, Università di Perugia, Via Elce di Sotto 8, 06123 Perugia, Italy.
*christian.ehm@unina.it

Table of content

- DFT results including long range Grimme-type dispersion corrections..2
- Measures of steric congestion at Al-bht sites..3
- Reaction schemes for sites A and B 4
- Ranges of NPA charges 5
- Reaction Gibbs free energies for py coordination after $AlMe_2R$ dissociation 5
- Final energies, entropy and enthalpy corrections 6
- Full Gaussian citation 8

- **DFT results including long range Grimme-type dispersion corrections**

Table S1. Calculated Δ*G* at 298 K (in kcal/mol) for the replacement of 'structural' $AlMe_3$ with 'structural' $AlMe_2$(bht) (Scheme 2a). At the RI-M06-2X(PCM)-D0/TZ//RI-TPSSTPSS/DZ.

Entry	Al-site	'Structural' $AlMe_3$ to 'structural' $AlMe_2$(bht)
1	**A**	-26.7
2	**B**	-32.4
3	**C**	-29.9

Table S2. Calculated Δ*G* at 298 K (in kcal/mol) for the release of $[AlMeR]^+$ or $AlMe_2R$ from MAO or MAO/BHT (Scheme 2b-d). At the RI-M06-2X(PCM)-D0/TZ//RI-TPSSTPSS/DZ.

Entry	Al-site	without donors			
		**$AlMe_3$ loss**	**$AlMe_2$(bht) loss**	**$[AlMe_2]^+$ loss**	**$[AlMe(bht)]^+$ loss**
1	**A**	26.4	18.8	105.8	95.6
2	**B**	37.5	35.5	97.8	93.3
3	**C**	9.2	4.8	76.5	69.5
		**with py**			
4	**A**	0.6	-6.7	24.0	19.6
5	**B**	-8.4	-9.9	16.0	17.3
6	**C**	-33.0	-37.1	-4.5	-5.6
		**with bipy**			
7	**A**	-	-	22.0	18.5
8	**B**	-	-	14.0	16.2
9	**C**	-	-	-6.5	-6.7

- **Measures of steric congestion at Al-bht sites**

To quantitatively describe of the distortion of the Al-O-Ph geometry, the Al-O-$C_{ipso,\ bht}$ angle and Al-O-$C_{ipso,\ bht}$-$C_{ortho,\ bht}$ dihedral were compared for sites **A**-**C** and for $AlMe_2$(bht), which is considered as reference species having minimum steric strain. The deviation from the optimal conformation is evident for **A** and less marked for **B-C**, especially in terms of Al-O-$C_{ipso,\ bht}$-$C_{ortho,\ bht}$ dihedral (Table S3; see also Figure S1).

Table S3. Al-O-$C_{ipso,\ bht}$ angle and Al-O-$C_{ipso,\ bht}$-$C_{ortho,\ bht}$ dihedral (in deg) for optimized geometries.

Entry	Al-site	Al-O-$C_{ipso,\ bht}$	Al-O-$C_{ipso,\ bht}$-$C_{ortho,\ bht}$
1	**A**	163	145
2	**B**	160	78
3	**C**	143	93
4	**$AlMe_2$(bht)**	125	97

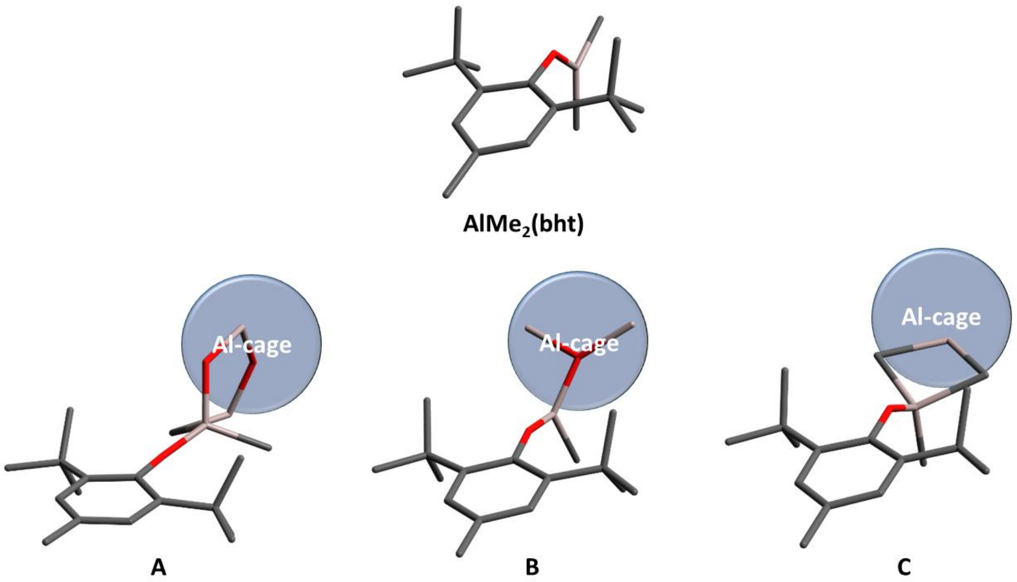

Figure S1. Comparison of optimized geometries for $AlMe_2$(bht) (top) and for the fragments of Al-cages bearing a bht group (bottom).

- **Reaction schemes for sites A and B**

a)

b)

c)

d)

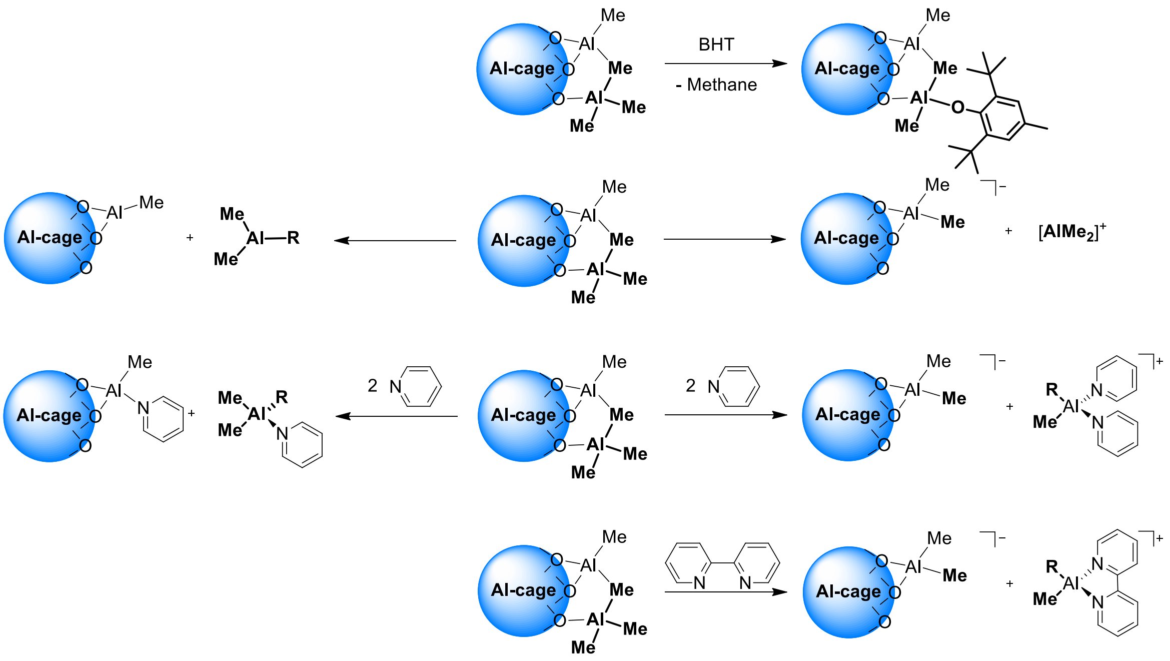

Scheme S1. Reactions studied in this work for type **A** Lewis acidic sites: a) replacement of 'structural' $AlMe_3$ with 'structural' $AlMe_2$(bht); b) release of $[AlMeR]^+$ or $AlMe_2R$ in the absence of neutral donors; c) release of $[AlMeR]^+$ or $AlMe_2R$ in the presence of **py**; d) release of $[AlMeR]^+$ in the presence of **bipy**. 'Structural' $AlMe_2R$ molecules highlighted in bold (R = Me or bht).

a)

b)

c)

d)

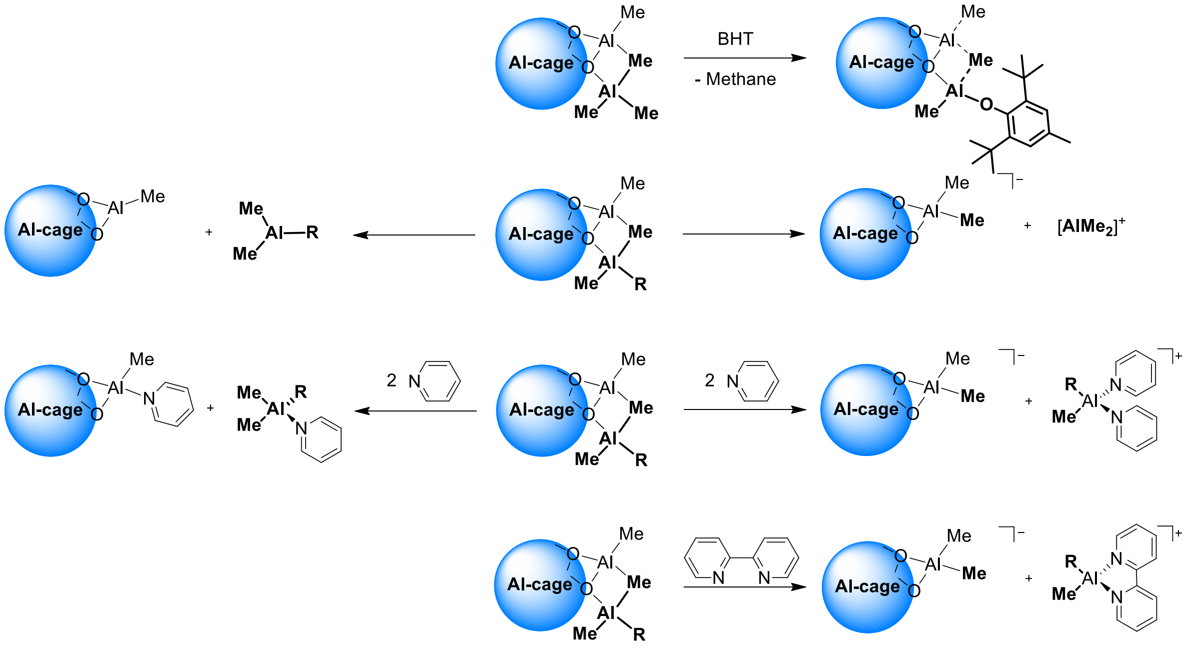

Scheme S2. Reactions studied in this work for type **B** Lewis acidic sites: a) replacement of 'structural' $AlMe_3$ with 'structural' $AlMe_2$(bht); b) release of $[AlMeR]^+$ or $AlMe_2R$ in the absence of neutral donors; c) release of $[AlMeR]^+$ or $AlMe_2R$ in the presence of **py**; d) release of $[AlMeR]^+$ in the presence of **bipy**. 'Structural' $AlMe_2R$ molecules highlighted in bold (R = Me or bht).

- **Ranges of NPA charges**

Table S4. Ranges of natural population analysis (NPA) charges on Al, O and C atoms of the anionic cages generated by $[AlMeR]^+$ dissociation from 16,6.

Entry	Al-site	Range of NPA charges		
		Al	O	C
1	**A**	0.40; 0.49	-0.46; -0.34	-0.25; -0.18
2	**B**	0.40; 0.51	-0.41; -0.34	-0.25; -0.18
3	**C**	0.36; 0.51	-0.36; -0.34	-0.26; -0.19
4*	**16,6**	0.41; 0.51	-0.36; -0.33	-0.24; -0.18

*Ranges of NPA charges for 16,6 reported for comparison.

- **Reaction Gibbs free energies for py coordination after $AlMe_2R$ dissociation**

Table S5. Calculated ΔG_R for py coordination to MAO after $AlMe_2R$ dissociation and from MAO, and $\Delta\Delta G_R$ for $AlMe_2R$ dissociation in the presence or absence of donor (see also Table2).

Entry	Al-site	py coordination		$AlMe_3$ loss		$AlMe_2$(bht) loss	
		ΔG_R	rel. ΔG_R	$\Delta\Delta G_R$	rel. $\Delta\Delta G_R$	$\Delta\Delta G_R$	rel. $\Delta\Delta G_R$
1	**A**	-5.7	0.0	-23.8	0.0	-22.8	0.0
2	**B**	-25.9	-20.2	-44.0	-20.0	-43.0	-20.0
3	**C**	-23.9	-18.2	-42.0	-18.2	-40.9	-18.2

At 298 K, in kcal/mol. rel. = relative.

- **Final energies, entropy and enthalpy corrections**

Table S6 - Final energies, entropy and enthalpy corrections (T=298 K, p=1.0 atm) in Hartree

Name	RI-TPSSTPSS/DZ					RI-M06-2X(PCM)/TZ	RI-M06-2X(PCM)-D0/TZ
	Formula	Energy	ZPE	EnthalpyCorr	EntropyCorr	Energy	Energy
CH4	CH4	-40.52284	0.04416	0.04798	0.02115	-40.50091	-40.50091
BHT_OH	C15H24O	-661.43934	0.34984	0.36958	0.06434	-661.21820	-661.22071
py	C5H5N	-248.35152	0.08710	0.09241	0.03212	-248.26267	-248.26277
bipy	C10H8N2	-495.51142	0.15453	0.16439	0.04411	-495.34113	-495.34175
AlMe3	C3H9Al	-362.22223	0.10391	0.11360	0.04457	-362.14957	-362.14984
AlMe3py	C8H14AlN	-610.61238	0.19393	0.20871	0.05583	-610.45768	-610.45872
AlMe2_+	C2H6Al	-322.02582	0.06978	0.07665	0.03585	-322.01667	-322.01677
AlMe2py2_+	C12H16AlN2	-818.89754	0.25051	0.26803	0.06317	-818.70215	-818.70388
AlMe2bipy_+	C12H14AlN2	-817.70409	0.22925	0.24541	0.05818	-817.50948	-817.51103
AlMe2BHT	C17H29AlO	-983.19221	0.40950	0.43562	0.08039	-982.92589	-982.92971
AlMeBHT+	C16H26AlO	-943.01501	0.37465	0.39782	0.07279	-942.79605	-942.79908
AlMe2BHTpy	C22H34AlNO	-1231.57584	0.49957	0.53109	0.09190	-1231.23256	-1231.23817
AlMeBHTpy2_+	C26H36AlN2O	-1439.85394	0.55539	0.59006	0.10110	-1439.47014	-1439.47718
AlMeBHTbipy_+	C26H34AlN2O	-1438.66129	0.53405	0.56726	0.09605	-1438.27613	-1438.28274
16_6	C34H102Al22O16	-7897.80347	1.29548	1.42543	0.29103	-7896.97494	-7897.00451
16_6_BHT_siteA	C48H122Al22O17	-8518.74865	1.60059	1.74765	0.32714	-8517.73554	-8517.77215
16_6_BHT_siteBa	C48H122Al22O17	-8518.75724	1.60085	1.74771	0.32690	-8517.74576	-8517.78148
16_6_BHT_siteBb	C48H122Al22O17	-8518.75271	1.60074	1.74767	0.32730	-8517.73624	-8517.77195
16_6_BHT_siteC1a	C48H122Al22O17	-8518.75863	1.60050	1.74749	0.32870	-8517.74111	-8517.77621
16_6_BHT_siteC1b	C48H122Al22O17	-8518.75634	1.60002	1.74724	0.32854	-8517.73892	-8517.77448
16_6_BHT_siteC2a	C48H122Al22O17	-8518.75717	1.60038	1.74748	0.32891	-8517.74053	-8517.77594
16_6_BHT_siteC2b	C48H122Al22O17	-8518.75887	1.60143	1.74796	0.32580	-8517.74202	-8517.77720
16_6_minusAlMeR_siteA_-	C32H96Al21O16	-7575.53123	1.22005	1.34449	0.28217	-7574.76959	-7574.79677
16_6_minusAlMeR_siteB_-	C32H96Al21O16	-7575.54714	1.22097	1.34450	0.27798	-7574.78450	-7574.81234
16_6_minusAlMeR_siteC1_-	C32H96Al21O16	-7575.56879	1.22031	1.34443	0.27969	-7574.81578	-7574.84382

16_6_minusAlMeR_siteC2_-	C32H96Al21O16	-7575.57238	1.22088	1.34485	0.27949	-7574.81760	-7574.84567
16_6_minusAlMe2R_siteA	C31H93Al21O16	-7535.55042	1.18807	1.30913	0.27358	-7534.76527	-7534.79167
16_6_minusAlMe2R_siteB	C31H93Al21O16	-7535.51964	1.18829	1.30940	0.27417	-7534.74715	-7534.77391
16_6_minusAlMe2R_siteC1	C31H93Al21O16	-7535.56663	1.18746	1.30889	0.27402	-7534.79141	-7534.81858
16_6_minusAlMe2R_siteC2	C31H93Al21O16	-7535.56590	1.18773	1.30911	0.27521	-7534.78949	-7534.81656
16_6_minusAlMe2R_siteA_py	C36H98Al21NO16	-7783.90344	1.27736	1.40418	0.28673	-7783.05243	-7783.08157
16_6_minusAlMe2R_siteB_py	C36H98Al21NO16	-7783.91913	1.27716	1.40401	0.28691	-7783.06635	-7783.09549
16_6_minusAlMe2R_siteC1_py	C36H98Al21NO16	-7783.95656	1.27805	1.40454	0.28500	-7783.10953	-7783.13893
16_6_minusAlMe2R_siteC2_py	C36H98Al21NO16	-7783.95559	1.27815	1.40465	0.28524	-7783.10693	-7783.13658
16_6_AlMeBHTbipy_IP	C58H130Al22N2O17	-9014.27656	1.75669	1.91438	0.35425	-9013.09688	-9013.13593

- **Full Gaussian citation**

Gaussian 09, Revision B.01, Frisch, M. J.; Trucks, G. W.; Schlegel, H. B.; Scuseria, G. E.; Robb, M. A.; Cheeseman, J. R.; Scalmani, G.; Barone, V.; Mennucci, B.; Petersson, G. A.; Nakatsuji, H.; Caricato, M.; Li, X.; Hratchian, H. P.; Izmaylov, A. F.; Bloino, J.; Zheng, G.; Sonnenberg, J. L.; Hada, M.; Ehara, M.; Toyota, K.; Fukuda, R.; Hasegawa, J.; Ishida, M.; Nakajima, T.; Honda, Y.; Kitao, O.; Nakai, H.; Vreven, T.; Montgomery, J. A., Jr.; Peralta, J. E.; Ogliaro, F.; Bearpark, M.; Heyd, J. J.; Brothers, E.; Kudin, K. N.; Staroverov, V. N.; Kobayashi, R.; Normand, J.; Raghavachari, K.; Rendell, A.; Burant, J. C.; Iyengar, S. S.; Tomasi, J.; Cossi, M.; Rega, N.; Millam, M. J.; Klene, M.; Knox, J. E.; Cross, J. B.; Bakken, V.; Adamo, C.; Jaramillo, J.; Gomperts, R.; Stratmann, R. E.; Yazyev, O.; Austin, A. J.; Cammi, R.; Pomelli, C.; Ochterski, J. W.; Martin, R. L.; Morokuma, K.; Zakrzewski, V. G.; Voth, G. A.; Salvador, P.; Dannenberg, J. J.; Dapprich, S.; Daniels, A. D.; Farkas, Ö.; Foresman, J. B.; Ortiz, J. V.; Cioslowski, J.; Fox, D. J. Gaussian, Inc., Wallingford CT, 2009.
